# Tendon Grafting: A New Operation for Deformities Following Infantile Paralysis, with Report of a Successful Case

**Published:** 1895-11

**Authors:** Samuel E. Milliken

**Affiliations:** New York, Surgeon in Chief of the New York Infirmary for Crippled Children; Surgeon to the Infants’ and Children’s Hospital; 640 Madison Avenue


### TENDON GRAFTING: A NEW OPERATION FOR DEFORMITIES FOLLOWING INFANTILE PARALYSIS, WITH REPORT OF A SUCCESSFUL CASE.

BY SAMUEL E. MILLIKEN, M D., NEW YORK,

Surgeon in Chief of the New York Infirmary for Crippled Children; Surgeon to the Infants' and Children's Hospital.

At the meeting of the New York State Medical Association, October 15th, 1895 (*Medical Record*, October 26th), Dr. Milliken presented a boy eleven years of age, upon whom, twenty months before, he had successfully grafted part of the extensor tendon of the great toe into the tendon of the tibialis anticus muscle, the latter having been paralyzed since the child was eighteen months old.

The case which was presented, showed the advantages of only taking part of the tendon of a healthy muscle which was made to carry on the function of its paralyzed associate, without in any way interfering with its own work.

The brace, which had been worn since two years of age, was left off, the patient walked without a limp. The talipes valgus was entirely corrected, and the boy had become quite an expert on roller skates.

Dr. Milliken predicts a great field for tendon grafting in these otherwise hopeless cases of infantile paralysis, who, heretofore, have been doomed to the wearing of braces all their lives. (Author's Abstract.)

*640 Madison Avenue, New York.*

---

BLEEDING HÆMORRHOIDS.—Order complete rest in horizontal position; bathe region with cold boracic lotion. If pain is acute, apply an ointment of vaseline, in each ounce of which are two grains of muriate cocaine, five grains extract belladonna, and seven and a half grains extract kramerir. If hemorrhage is severe, apply a solution of iron perchloride on cotton wool. Reduce hemorrhoids with sponge soaked in cold water. In the evening, introduce a suppository containing one-fifth grain of extract belladonna, one-half grain of extract opium, fifteen grains of extract krameria, and sixty grains of cocoa butter. If the hemorrhoids continue to cause annoyance, surgical interference, either by forced dilatation of the sphincter or by extirpation, will be necessary.—*Practitioner.* (London).

